# Facial asymmetry tracks genetic diversity among *Gorilla* subspecies

**DOI:** 10.1098/rspb.2021.2564

**Published:** 2022-02-23

**Authors:** Kate McGrath, Amandine B. Eriksen, Daniel García-Martínez, Jordi Galbany, Aida Gómez-Robles, Jason S. Massey, Lawrence M. Fatica, Halszka Glowacka, Keely Arbenz-Smith, Richard Muvunyi, Tara S. Stoinski, Michael R. Cranfield, Kirsten Gilardi, Chantal Shalukoma, Emmanuel de Merode, Emmanuel Gilissen, Matthew W. Tocheri, Shannon C. McFarlin, Yann Heuzé

**Affiliations:** ^1^ State University of New York, College at Oneonta, Oneonta, NY 13820, USA; ^2^ Univ. Bordeaux, CNRS, MC, PACEA, UMR 5199, 33615, Pessac, France; ^3^ Center for the Advanced Study of Human Paleobiology, Department of Anthropology, The George Washington University, Washington, DC 20052, USA; ^4^ Department of Biology, University of Indianapolis, 46227, USA; ^5^ Physical Anthropology Unit, Department of Biodiversity, Ecology, and Evolution, Faculty of Biological Sciences, Complutense University of Madrid, Madrid, Spain; ^6^ Department of Clinical Psychology and Psychobiology, University of Barcelona, Passeig de la Vall d'Hebron 171, 08035 Barcelona, Spain; ^7^ Department of Anthropology, University College London, 14 Taviton St, London WC1H 0BW, UK; ^8^ Department of Anatomy and Developmental Biology, Biomedicine Discovery Institute, Monash University, Clayton, Victoria 3800, Australia; ^9^ Department of Physiological Sciences, University of Florida College of Veterinary Medicine, Gainesville, FL, USA; ^10^ Department of Basic Medical Sciences, University of Arizona College of Medicine-Phoenix, Phoenix 85004, USA; ^11^ Department of Small Animal Clinical Sciences, Virginia-Maryland College of Veterinary Medicine, Virginia Tech, Blacksburg, VA 24061, USA; ^12^ Department of Tourism and Conservation, Rwanda Development Board, Kigali, Rwanda; ^13^ The Dian Fossey Gorilla Fund International, Atlanta, GA 30315, USA; ^14^ Gorilla Doctors (MGVP, Inc.), Karen C. Drayer Wildlife Health Center, University of California Davis, Davis, CA 95616, USA; ^15^ Institut Congolais pour la Conservation de la Nature, Virunga National Park, Rumangabo, Democratic Republic of Congo; ^16^ Department of African Zoology, Royal Museum for Central Africa, Tervuren, Belgium; ^17^ Laboratory of Histology and Neuropathology, Université Libre de Bruxelles, Brussels, Belgium; ^18^ Department of Anthropology, Lakehead University, Thunder Bay, Ontario, Canada P7B 5E1; ^19^ Human Origins Program, National Museum of Natural History, Smithsonian Institution, Washington, DC 20013, USA; ^20^ Australian Research Council Centre of Excellence for Australian Biodiversity and Heritage, University of Wollongong, Wollongong, New South Wales 2522, Australia

**Keywords:** asymmetry, great apes, geometric morphometrics, inbreeding, stress

## Abstract

Mountain gorillas are particularly inbred compared to other gorillas and even the most inbred human populations. As mountain gorilla skeletal material accumulated during the 1970s, researchers noted their pronounced facial asymmetry and hypothesized that it reflects a population-wide chewing side preference. However, asymmetry has also been linked to environmental and genetic stress in experimental models. Here, we examine facial asymmetry in 114 crania from three *Gorilla* subspecies using 3D geometric morphometrics. We measure fluctuating asymmetry (FA), defined as random deviations from perfect symmetry, and population-specific patterns of directional asymmetry (DA). Mountain gorillas, with a current population size of about 1000 individuals, have the highest degree of facial FA (explaining 17% of total facial shape variation), followed by Grauer gorillas (9%) and western lowland gorillas (6%), despite the latter experiencing the greatest ecological and dietary variability. DA, while significant in all three taxa, explains relatively less shape variation than FA does. Facial asymmetry correlates neither with tooth wear asymmetry nor increases with age in a mountain gorilla subsample, undermining the hypothesis that facial asymmetry is driven by chewing side preference. An examination of temporal trends shows that stress-induced developmental instability has increased over the last 100 years in these endangered apes.

## 1. Background

Facial symmetry is widely regarded as a reliable indicator of attractiveness and reproductive success in humans, while asymmetry is often used as a measure of early life stress [1,2]. As both sides of bilaterally symmetric faces share the same genotype, it is expected that they will exhibit the same phenotype, except when individuals experience instability during development [2]. Therefore, studies typically use fluctuating asymmetry (FA) as a measure of individual or population-level fitness, calculated as the random deviations from perfect symmetry or from population-specific patterns of directional asymmetry (DA) [2,3]. Most FA studies have focused on elucidating the environmental causes, although there is also evidence suggesting that FA is heritable [4,5]. Experimental studies have linked environmental stressors and inbreeding to the level of FA in bilateral structures of rodents and flies [6–8]. In humans, it has been suggested that genetic or environmental stress increases susceptibility to health problems later in life, such that FA might provide a reliable signal of fitness [9]. As a result, studies measuring FA in non-human primate faces have focused on the link between FA and adult fitness or health outcomes [10], and not necessarily the environmental conditions under which individuals developed. As such, the possible stressors behind facial FA beyond the classical ‘environmental or genetic' dichotomy remain poorly understood. Moreover, surprisingly little is known about the evolutionary significance of facial asymmetry, including the magnitude of facial FA in extinct hominins and our closest living relatives, the non-human apes [11,12].

**Figure 1.**
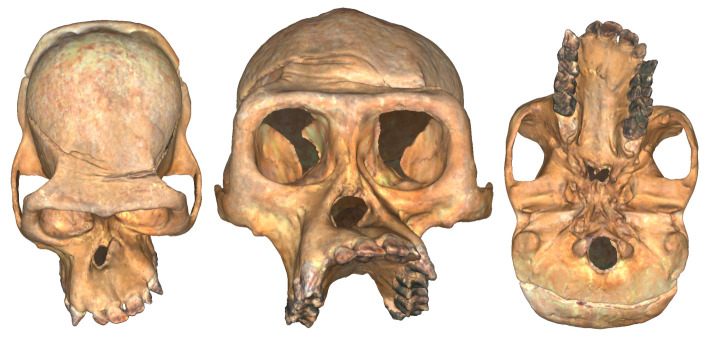
An extreme example of facial asymmetry in a female Virunga mountain gorilla cranium (Tayna, individual GP.148), shown as a three-dimensional surface model with texture. This individual was not included in the sample because she was dentally immature at the time of death, but she exhibits an extreme version of the asymmetric pattern documented in this study. (Online version in colour.)

Groves & Humphrey [13] first described the marked asymmetry present in the craniofacial skeletons of Virunga mountain gorillas (*Gorilla beringei beringei*) studied at the Dian Fossey Gorilla Fund's Karisoke Research Center (figure 1). They found that western lowland gorilla (*G. gorilla gorilla*) faces were not significantly asymmetric (*n* = 138), but 19 of 55 eastern gorillas (i.e. mountain and Grauer (*G. beringei graueri*)) had faces that were at least 4 mm longer on the left side than the right, and of these individuals, 18 were Virunga mountain gorillas. Mountain gorillas were the only subspecies with almost as many asymmetric as symmetric individuals in the sample, and the authors suggested that the observed asymmetry may reflect a preference for chewing on the left side [13]. This ‘gross asymmetry' was further evidenced by the presence of uneven tooth wear and lopsidedness of the sagittal crest, but they acknowledged that other closely related taxa with equally developed masticatory musculature, such as orangutans, did not seem to show similar levels of facial asymmetry. Indeed, while habitual unilateral chewing is commonly invoked as an explanation for facial asymmetry [14], this link has largely been assumed rather than tested.

**Figure 2.**
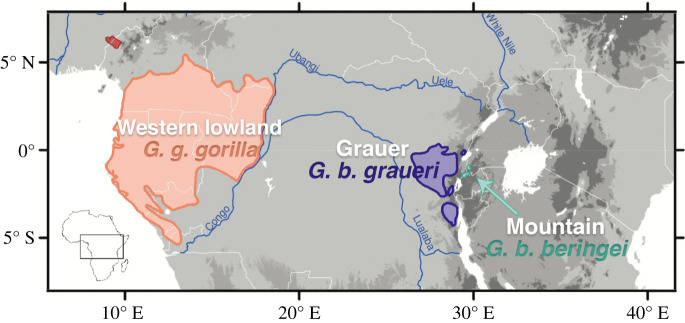
The approximate ranges of the four *Gorilla* subspecies. The three subspecies analysed in this study are labelled (western lowland gorillas, Grauer gorillas and mountain gorillas), while cross river gorillas (*G. g. diehli*) are shown in red at the top left corner of the map. Major river boundaries (blue), elevation grades (grey) and country borders (white) are also shown. Modified from Tocheri *et al*. [15]. (Online version in colour.)

In this study, we use three-dimensional geometric morphometrics to quantify adult facial skeleton asymmetry in three gorilla subspecies with well-documented variation in environmental (extrinsic) and genetic (intrinsic) stress, namely western lowland gorillas, Grauer gorillas and Virunga mountain gorillas (figure 2). Although we are not directly measuring genetic or environmental variables, we use the term ‘stress' to describe the factors hypothesized to increase developmental instability, thus leading to measurable facial asymmetry in the skeleton. In addition to FA, we also analyse DA, which occurs when one side differs consistently from the other at the population level, in line with the differential chewing hypothesis proposed by Groves & Humphrey [13]. We test whether the marked asymmetry in mountain gorillas is significantly greater than that expressed by other gorilla taxa, and evaluate the results considering current ecological, behavioural, and genetic information. Because asymmetric variation usually only explains a small proportion of the total variation in morphological analyses, we also characterize symmetric variation in facial morphology, as well as subspecies-level variation in facial asymmetry as it relates to sexual dimorphism. We investigate three alternative hypotheses to test whether genetic stress, environmental stress, or chewing side preference better correspond with facial asymmetry among gorilla subspecies.

First, we test whether more inbred gorilla subspecies exhibit more pronounced facial FA. In terms of genetic stress or inbreeding, mountain gorillas are homozygous at about one third of their genomes, and thus have very low genetic diversity compared to western lowland and, to a lesser extent, Grauer gorillas [16,17]. Mountain gorillas are more inbred than even the most inbred contemporary human populations [18] and the Altai Neanderthal [19]. While several studies have suggested that inbreeding might lead to higher levels of FA in model species [8], this study provides an opportunity to assess facial FA in the case of extreme inbreeding yet relatively stable socioecological conditions of the mountain gorilla.

Second, we assess whether gorillas that experience more environmental stress exhibit more pronounced facial FA. While environmental stress encompasses many factors, one main axis of variation among gorilla subspecies lies in dietary and ecological variability, with western lowland gorillas being exposed to the highest level of seasonal unpredictability in food resources and competition [20]. Western lowland gorillas have also experienced major population declines due to human activity and infectious diseases, most notably Ebola, resulting in approximately 90% casualties in affected populations [21]. By contrast, Virunga mountain gorillas eat a reliable, almost entirely folivorous diet, and have experienced increased population growth over the past several decades [22]. Grauer gorillas fall between these two extremes, with those from highland areas eating a more folivorous diet, and those from lowland areas eating a similar proportion of fruit as western lowland gorillas [20,23]. Grauer gorillas have also experienced major population declines in the last century, losing up to 77% of their total population [24]. In terms of gorilla behavioural ecology, these dietary and other social differences form potential sources of variation in environmental stress among taxa.

Third, we test whether gorillas exhibit more pronounced facial asymmetry because they have a chewing side preference. If gorillas preferentially chew on one side of the mouth, then they can be expected to show differences in the degree of tooth wear between the left and the right sides, which will match the pattern in facial asymmetry. A subsample of Virunga mountain gorillas with associated tooth wear data are used to test whether they exhibit chewing side preferences as evidenced by uneven tooth wear, and the patterns of facial asymmetry are considered in light of those results.

## 2. Material and methods

The sample includes the crania of 40 Virunga mountain gorillas (*Gorilla beringei beringei*), 40 Grauer gorillas (*G. beringei graueri*) and 34 western lowland gorillas (*G. gorilla gorilla*), with equal representation of females and males. We analysed adult individuals as determined by the third permanent molar being fully erupted and in occlusion. Only those with completely preserved facial anatomy, and no clear evidence of trauma or pathology, were included. In the mountain gorilla sample, 22 of the individuals were from the Mountain Gorilla Skeletal Project (Musanze, Rwanda) and three-dimensional models were digitized with a Breuckmann SmartScan white light scanner (aligned and merged in Optocat software v.11.01.06-2206). The remaining 92 models were reconstructed from medical CT scans at the Katholieke Universiteit Leuven, Belgium (Siemens Sensation 64, 120 kV, 135 mA, 1 mm slice thickness, reconstruction interval of 0.5 mm, 15 cm field of view, 0.29296875 mm pixel size, 512*512 pixel matrix) [25], and the Smithsonian's National Museum of Natural History (Washington, DC; Siemens Somatom Emotion CT Scanner, 110 kV, 70 mA, 1 mm slice thickness, 0.1 mm reconstruction increment; surface models generated in Materialize Mimics). A recent study suggests that there are no significant differences in models derived from different imaging modalities [26], allowing for the direct comparisons made here.

We used Viewbox 4 software (http://www.dhal.com/) to place 156 homologous landmarks and curve sliding semilandmarks on the cranial models (electronic supplementary material, figure S1). The landmark configuration uses classic fixed facial landmarks supplemented by curve sliding semilandmarks set on the face and palate. Because of the uncertainty in semilandmark location, they were slid along their corresponding curves with respect to the fixed landmarks in order to minimize bending energy following a standard procedure for semilandmark analyses [27]. Landmarks were digitized twice on each individual to assess FA and parse it out from measurement error via Procrustes ANOVA, as described below. The raw three-dimensional coordinates were subjected to Procrustes superimposition to remove the effect of scale, orientation and position from the shape analyses [28]. The symmetric versus asymmetric components of shape variation were then analysed separately: the symmetric component comprised the original and mirrored landmark configurations for each cranium, while the asymmetric component includes deviations of the original configurations from the symmetric averages [29–32]. Principal component analysis was conducted to analyse the main patterns of variation using symmetric coordinates and asymmetric residuals. The effects of allometry were assessed using a multivariate regression of shape versus centroid size. Analyses of the different components of variance were conducted using Procrustes ANOVA [29], in which the factor ‘individual' represents symmetric variation, ‘side' represents one-sided or DA, and the interaction between the two represents non-directional or FA. Measurement error was calculated as the residual variation in the Procrustes ANOVA, and explains between 1.6 and 2.2% of the shape variation (table 1).

**Table 1.** Interspecific Procrustes ANOVAs of gorilla facial morphology. SS, sum of squares; MS, mean squares (multiplied by 1000); d.f., degrees of freedom; *F*, *F*-ratio; *p*: *p*-value; %var, percentage of variance explained by each effect; S, symmetry; DA, directional asymmetry; FA, fluctuating asymmetry. Asterisks mark significant differences among taxa, as determined by 1000 bootstraps of observed FA correlation matrices (see electronic supplementary material, table S3 for details).

taxon	effect	SS	MS*	d.f.	*F*	*p*	%var
mountain gorillas (*G.b.b.*)	individual (S)	0.2178	0.240	9087	4.61	**<0.001**	**79.1**
*n* = 40	side (DA)	0.0055	0.242	228	4.65	**<0.001**	**2.0**
	ind × side (FA)	0.0463	0.052	8892	19.99	**<0.001**	16.8*
	measurement error	0.0048	0.003	18 440			**1.7**
	**total**	**0.2755**					
Grauer gorillas (*G.b.g.*)	individual (S)	0.2254	0.248	9087	9.92	**<0.001**	**88.1**
*n* = 40	side (DA)	0.0015	0.067	228	2.69	**<0.001**	**0.6**
	ind × side (FA)	0.0222	0.025	8892	8.07	**<0.001**	8.7*
	measurement error	0.0057	0.003	18 440			**2.2**
	**total**	**0.2559**					
western lowland gorillas (*G.g.g.*)	individual (S)	0.2123	0.276	7689	14.54	**<0.001**	**91.1**
*n* = 34	side (DA)	0.0017	0.074	228	3.91	**<0.001**	**0.7**
	ind × side (FA)	0.0143	0.019	7524	7.93	**<0.001**	6.1*
	measurement error	0.0038	0.002	15 674			**1.6**
	**total**	**0.2331**					

To test for differences in the magnitude of facial FA among taxa, we conducted a bootstrapping analysis of the correlation matrices for the FA component following Webster & Zelditch [33]. The probability of matrices being identical was assessed by 1000 bootstraps (unpublished R code from Haber, provided by Webster & Zelditch [33]). Individual facial asymmetry scores were calculated with respect to a perfectly symmetric configuration to measure the magnitude of asymmetry across the whole face. This was done by calculating the Procrustes distance between the original and reflected and relabelled landmark configurations following Procrustes registration [2]. Individual asymmetry scores were compared to the magnitude of tooth wear asymmetry because there was no clear population-level chewing side preference (figure 4). If there had been a clear directional signal in tooth wear, directional facial asymmetry would have been the more appropriate form of asymmetry to assess in relation to tooth wear.

In a subsample of Virunga mountain gorillas (sample sizes specified in each table and figure), we assessed the relationships between tooth wear asymmetry in upper and lower molars of the same position (electronic supplementary material, table S4), facial asymmetry scores and molar wear asymmetry (electronic supplementary material, table S5; figure 4), the relationship between each variable and age at death (electronic supplementary material, figure S2), and facial asymmetry scores through time (figure 4) using Spearman's rank correlation analysis. We compared the magnitude and direction of tooth wear in upper versus lower molars of the same position as a test of whether this metric is consistent and a reliable indicator of chewing side preference. Tooth wear was assessed by calculating the per cent of dentine exposure in the first permanent molars following Galbany *et al*. [34]. The percentage of the occlusal surface with exposed underlying dentine, relative to the total area of the occlusal surface, was measured in both the right and left first, second and third molars using digital photographs of original teeth. While a three-dimensional topographic measure of tooth wear, such as slope, is more sensitive to early stages of tooth wear [35], dentine becomes exposed on the M1 in mountain gorillas by the time the M3 erupts [34], and thus percentage of exposed dentine is sensitive enough to capture molar wear in this adult sample of gorillas. Known ages at death are available for a majority of the mountain gorilla individuals included in the subsample, but those without known ages were estimated based on incisor wear following the protocol developed by Galbany *et al*. [36], which estimates ages at death within about a 1–3 year error margin (see electronic supplementary material, figure S2 for sample details). The collection dates were known for most individuals in the sample, but in cases where there were several years in which remains were estimated to have been collected, we used the earliest possible date for our analysis. For example, 20 Grauer gorillas were collected between 1980 and 1984, so we used 1980. Ten mountain gorillas were also collected in estimated windows of 5 years (4 individuals) and 9 years (5 individuals), with one individual only being known to have been collected before 2001, so we substituted the year 2000 in that instance. Besides the known individuals with uncertain collection dates, there is likely some additional variation in record keeping across institutions, as well as collection practices over time, justifying our estimation and inclusion of the uncertain dates in this study. To assess the trend in facial asymmetry magnitude through time, we conducted a linear mixed model with collection date as a fixed effect and sex class (sex and subspecies) as a random effect in order to control for differences in the level of asymmetry among groups.

Analyses were carried out in MorphoJ and R (v. 4.0.0) [37].

## 3. Results

**Figure 3.**
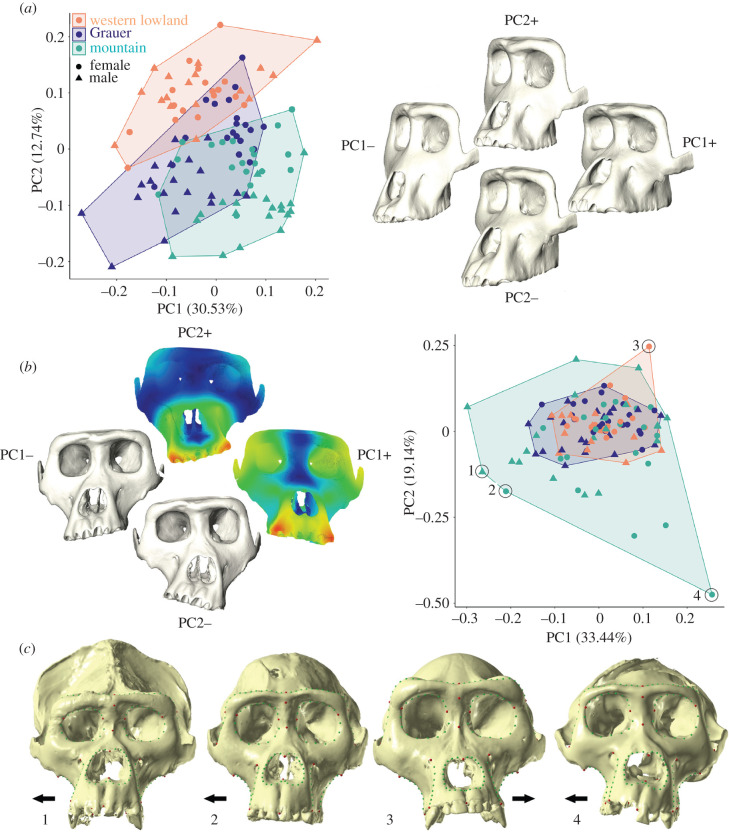
Facial shape variation in gorillas. (*a*) Principal component analysis (PCA) of the symmetric component of facial shape variation. Three-dimensional surface models of the mean shape warped along the positive and negative PC1 and PC2 axes. (*b*) PCA of the asymmetric component, with three-dimensional models of the mean shape warped along the positive and negative axes. Two models show distance-based heatmaps of the intensity of asymmetry across the face with red indicating more asymmetry and blue indicating less asymmetry. In the PCA, specific individuals are circled and shown in panel (*c*), which depicts actual surface models of the most asymmetric individuals. Arrows denote directionality of asymmetry in the lower midface. Green and red points represent fixed and sliding semilandmarks used in this study, respectively. (Online version in colour.)

The principal component analyses (PCAs) and Procrustes ANOVAs of the symmetric (i.e. the original and mirrored landmark configurations for each cranium) and asymmetric (i.e. deviations of the original configurations from the symmetric averages) aspects of shape variation show that 79–91% of shape differences within and among gorilla taxa are related to symmetric variation in facial morphology (figure 3*a*, table 1). The plot of the first (PC1) and second (PC2) principal components of the symmetric aspect, which explain 30.5% and 12.7% of the variance, respectively, shows separation of western lowland and mountain gorillas within the morphospace, mainly along PC2, with Grauer gorillas falling in between but overlapping more with mountain gorillas (figure 3*a*). In general, western lowland gorillas have relatively narrower, less prognathic faces with more rounded orbits framed by a curved supraorbital torus. By contrast, mountain gorillas have relatively broader, more prognathic faces and more rectangular orbits framed by a flat supraorbital torus. Grauer gorillas have the narrowest faces of the three taxa, rounder orbits, and a taller nasal aperture and rostrum. When sex is considered, male and female mountain gorillas separate primarily along PC2, while male and female Grauer gorillas separate along PC1 and PC2 (figure 3*a*). By contrast, male and female western lowland gorillas do not clearly separate along either of the first two PCs (figure 3*a*), nor PC3 (not shown). The multivariate regression of shape against size indicates that allometry explains about 10% of the shape variation in the symmetric aspect (*r* = 0.10; *p* < 0.001), and 15% (*r* = 0.38; *p* < 0.001) and 8% (*r* = 0.28; *p* = 0.002) for PC1 and PC2, respectively.

**Figure 4.**
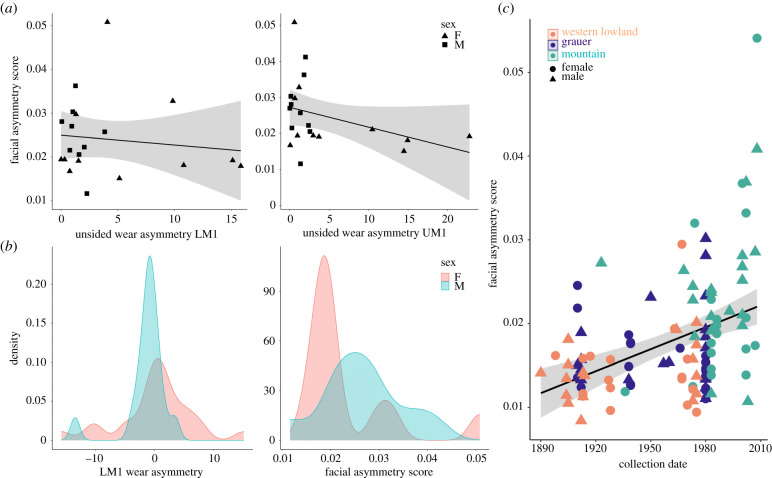
(*a*) Regressions of unsided tooth wear asymmetry in lower first molars (LM1, *n* = 20) (*r_s_* = –0.19, *p* = 0.422) and upper first molars (UM1, *n* = 21) (*r_s_* = –0.43, *p* = 0.054) versus facial asymmetry scores in the Virunga mountain gorilla subsample. Shading shows 95% confidence intervals. (*b*) Density plots of tooth wear asymmetry of LM1 (*n* = 41) and facial asymmetry scores (*n* = 22) in Virunga mountain gorillas. At left, positive values indicate greater LM1 wear on the right side, while negative values indicate greater LM1 wear on the left side. At right, larger numbers denote greater facial asymmetry overall. (*c*) Facial asymmetry scores of individual western lowland, Grauer and mountain gorillas (*n* = 114). Facial asymmetry increases through time when controlling for sex and species differences in the magnitude of asymmetry (*F*_1,107_ = 4.95, *p* = 0.028). Grey shading shows 95% confidence intervals. (Online version in colour.)

The PCA of the asymmetric aspect of shape shows that the range of variation in mountain gorillas envelops that of the other two taxa (figure 3*b*). Both DA and FA are highly significant in all three taxa (table 1), but FA explains a much larger proportion of variation, ranging from 6% in western lowland gorillas, to 9% in Grauer gorillas, and 17% in mountain gorillas compared to the 0.6–2.0% of shape variation explained by DA. The probability of observed FA correlation matrices being identical between each of the three taxa, as assessed by 1000 bootstraps, is 0, suggesting that mountain gorillas have significantly greater facial FA than both Grauer and western lowland gorillas, and that Grauer gorillas have significantly greater FA than western lowland gorillas (table 1; electronic supplementary material, table S3). To assess whether these results are related to the drastic reduction of gorilla habitat and population sizes, the collection years of the individuals (ranging from 1880 to 2008) were compared to facial asymmetry scores. The magnitude of facial asymmetry for each individual increases through time within the combined sample, even when controlling for sex and species differences in the magnitude of asymmetry (*F*_1,107_ = 4.95, *p* = 0.028). The most recent mountain gorillas exhibit the highest facial asymmetry scores of all (figure 4).

Though statistically significant in all three taxa, DA only explains a small percentage of morphological variation (0.6–2.0%), suggesting that it is not a major factor shaping facial morphology at the population level in gorillas (table 1). In all three taxa, most of the total asymmetry occurs in the lower midface, as shown in several examples of particularly asymmetric individuals (figure 3*c*), and in distance-based heatmaps summarizing the asymmetry of the whole sample (figure 3*b*). In the Virunga mountain gorilla subsample, we found that the upper and lower molars (UM and LM) of the same position (i.e. first, second or third) exhibit matching unsided wear (electronic supplementary material, table S4), but there is no relationship between unsided tooth wear asymmetry (i.e. considering only the magnitude) and facial asymmetry scores (electronic supplementary material, table S5, figure 4). In other words, individuals with the highest degree of differential wear between the left and the right side do not show the highest level of facial asymmetry.

Male Virunga mountain gorillas, which tend to be younger than females in this subsample, show less variation in right–left tooth wear differences compared to females (figure 4). However, there is no evidence of chewing side preference as inferred from tooth wear asymmetry within this population; distributions of tooth wear asymmetry centre close to 0 in all six teeth examined (figure 4 shows density plot for LM1). Older individuals exhibit more asymmetric tooth wear compared to younger individuals (*r_s_* = 0.56, *p* < 0.001 for LM1; *r_s_* = 0.66, *p* < 0.001 for UM1) (electronic supplementary material, figure S2), but there is no relationship between individual facial asymmetry scores and age (*r_s_* = 0.10, *p* = 0.694) in the Virunga mountain gorilla subsample (electronic supplementary material, figure S2).

## 4. Discussion

Bilateral asymmetry is used as a reliable indicator of developmental instability in humans despite the relative paucity of evidence linking it to specific early life stressors in primates or other long-lived mammals [2]. This study demonstrates that in gorillas, facial asymmetry mirrors known variation in genetic diversity, with the markedly inbred mountain gorillas exhibiting significantly more facial asymmetry than both Grauer and western lowland gorillas. Our results show that gorilla facial asymmetry is dominated by FA rather than DA due to chewing side preference as was originally suggested by Groves & Humphrey [13]. In the absence of population-level trends in lateralized tooth wear (figure 4), the lack of relationship between facial asymmetry scores and first molar tooth wear asymmetry in the markedly asymmetric mountain gorilla subsample suggests that facial asymmetry does not relate to preferential mastication on one side of the mouth, neither as a cause nor a consequence.

Intraspecific genetic diversity is increasingly recognized as a requirement for the long-term survival of species, but the genetic health of a population is difficult to assess without a major investment in genomic analyses [38]. Population fragmentation and reduction in population size are the driving forces behind reductions in diversity, with genetic drift becoming the dominant evolutionary force rather than selection [39]. All extant gorilla subspecies, three of which were analysed here, are either endangered or critically endangered because of infectious disease, hunting by humans, and habitat loss and degradation [40,41]. A consequence of reduced genetic diversity is an increase in deleterious mutations, threatening long-term population survival and even extinction through decreased fertility, reduced ability to adapt to environmental changes and susceptibility to disease [16,24].

Among the ancestors of eastern gorillas, at least one genetic bottleneck was followed by subsequent inbreeding over the last 100 000 years [16], probably resulting in high frequencies of otherwise rare hand and foot morphology [15] and temporalinsular fusions in the brain [42] within extant populations. Only about 1000 mountain gorillas, including both the Virunga and Bwindi populations, remain after experiencing long-term, sustained population declines. These declines have led to a host of issues including webbed feet and fertility problems [16], although the Virunga population size in particular has increased for several decades following successful conservation interventions [22]. By contrast, western lowland gorillas have the largest range and population size at around 350 000 individuals [43]. Grauer gorillas, endemic to the eastern Democratic Republic of Congo, have experienced a startling 70% population decline in the last 100 years due to factors like human encroachment and poaching, with only about 3800 individuals alive today [41]. The genetic diversity of Grauer gorillas is intermediate between mountain and western lowland gorillas, with lower genetic diversity in peripheral versus core groups, suggesting a strong effect of genetic drift and limited gene flow among small, isolated forest fragments [24]. However, Grauer gorillas are significantly more inbred than western lowland gorillas and much more like mountain gorillas in their level of genetic diversity, but without necessarily sharing the same level of facial asymmetry. It is possible that the collection dates of the Grauer gorillas sampled here may influence our results as inbreeding has likely increased dramatically in the time since the current sample was collected (figure 4). Further, the Virunga mountain gorilla sample analysed here exhibits lower genetic diversity than the only other population of mountain gorillas from Bwindi National Park, Uganda [17,24]. Future work should prioritize the assessment of changes in facial asymmetry through time, and where possible in the context of long-term field sites, consider population-level group dynamics and familial relationships as they relate to asymmetry.

In addition to increasing the number of deleterious genes, inbreeding also makes individuals more susceptible to developmental perturbations caused by environmental stress [2]. Short-term activation of the stress response helps vertebrates cope with fluctuating environmental conditions, but chronically elevated glucocorticoid levels are pathogenic in the sense that they deplete energy reserves, negatively affecting health, immunity, fertility and survival [44]. In mountain gorillas, and especially the Virunga population, the increase in deleterious mutations in genes important for immune function has probably reduced their resilience to environmental change and pathogen evolution [16]. The precise mechanism behind the patterns of asymmetry documented in this study are not yet clear, but environmental stress alone is unlikely to explain variation in FA among gorillas. Virunga mountain gorillas rely on an almost entirely folivorous diet that is both spatio-temporally abundant and high in protein, while western lowland gorillas rely most heavily on unpredictable, seasonal fruit in addition to herbs and leaves. The western lowland gorilla diet is comparatively lower in protein, but high in non-protein energy during periods of high fruit consumption [20,23,45]. When fruit-feeding, gorillas spend more time travelling and less time resting [45], which, in addition to competition with other apes and the comparative unpredictability of their ecological conditions, might provide differential sources of environmental stress. In terms of temporal trends, studies of brain size [46] and dental defect severity [47] suggest that Virunga mountain gorillas that died between the 1960s and 1980s were more developmentally stressed than those that died between the 1990s and 2010s, with smaller brain sizes and more severe enamel defects on their teeth. However, these previous studies had more limited timeframes of analysis (i.e. 1960s to 2010s), so further work would benefit from incorporating multiple stress indicators to better understand their relationships and what factors influence their development.

Clinical and experimental studies of facial FA have identified the lower midface and mandible as the regions with the most asymmetry, particularly in inbred individuals [8,36], in line with our results (figure 3). Lacy & Horner [8] proposed, based on their analysis of inbred rats, that asymmetry is a threshold phenomenon with no lessening impact of inbreeding on FA after generations of breeding. Like mountain gorillas, the inbred Australian wild rats also exhibit abnormalities of the digits in addition to pronounced lower midfacial asymmetry. In the clinical setting, Al Kaissi *et al*. [48] documented a connection between persistent torticollis of a congenital origin and facial asymmetry in humans due to the malformation of the atlas in three family members. Individuals with congenital torticollis usually exhibit hemihypoplasia in the midfacial skeleton, on the opposite side of the palsied sternocleidomastoid muscle, presenting as unilateral facial compression [49]. As shown in figures 1 and 3, the most asymmetric mountain gorillas show evidence of facial compression and hemihypoplasia in the midface, leading to the dramatic asymmetry of the lower partition (i.e. inferior to the lower margin of the nasal aperture). While we do not suggest torticollis as a mechanistic explanation for the marked asymmetry present in mountain gorillas, we highlight the commonalities between inbreeding and mid to lower facial asymmetry with a hinge-like compression of the midface. Further studies undertaken in well-documented hominoid populations should help to shed light on the mechanisms behind the development of pronounced facial FA in humans and non-human primates.

Assessing facial asymmetry in closely related yet differently adapted gorilla taxa provides a critical context with which to better interpret such features in past human populations. Asymmetry is almost always removed from morphometric analyses of fossils as it is typically assumed to be caused by deformation via taphonomic processes, but it might be worthwhile to revisit reconstruction methodologies to allow for the measurement of FA. Particularly when studying samples without associated genetic data, facial FA might be used as a proxy for inbreeding, which reduces long-term adaptability, survival and fitness [38]. Baab & McNulty [11] documented asymmetry in contemporary humans, non-human great apes and fossil hominins, and they showed that the infraorbital foramina, alare and lingual canine margins are by far the most asymmetric, matching the tendencies in the extant species documented here (figure 3). The shape of the facial skeleton of extant great apes is well documented as it is often used to aid in fossil reconstructions and phylogenetic interpretations, but it is worth considering whether extant hominoids are reliable models for such reconstructions [50,51], especially in light of the increase in facial asymmetry in the last 100 years.

Intraspecifically, the facial skeletons of western lowland gorillas are sexually dimorphic, with facial growth continuing longer after reaching dental maturity in females compared to males, ultimately decreasing the level of dimorphism in older age classes [52]. This continued growth is unlikely to result from biomechanical processes related to mastication as the changes are not centred around the lower face [52], unlike the asymmetry results presented in this study. Debate surrounds whether FA is expected to be higher or lower in faster-growing groups with shorter developmental windows compared to slower-growing, longer-lived ones [53]. Here, mountain gorillas are the faster-growing taxon, and within species, females exhibit slower growth rates and males later ages at sexual maturity [46,54]. Our results from the Virunga mountain gorilla subsample show that there is no relationship between facial asymmetry magnitude and age, suggesting that asymmetry primarily develops during ontogeny and remains relatively stable throughout adulthood (electronic supplementary material, figure S2). By contrast, tooth wear asymmetry continues to increase with age (electronic supplementary material, figure S2). The females in this sample are, on average, older than the males, reflecting the demographics of mountain gorillas. The greater variation in tooth wear asymmetry among females is likely a consequence of these age differences between the sexes (figure 4; electronic supplementary material, figure S2). Our facial asymmetry results do not clarify the contribution of the fetal environment versus later in life correction to facial asymmetry, but this should be further analysed in the context of documented ontogenetic samples. Other parts of the skeleton besides the face should also be examined for asymmetry.

## 5. Conclusion

Taken together, our study shows that pronounced facial asymmetry occurs in the most genetically stressed gorillas and that it is not obviously related to lateralized mastication. While the plight of mountain gorillas is well known, these methods may serve the conservation efforts of less well-studied species without available genomic data. Our results also show that facial asymmetry has increased through time in all three gorilla subspecies, suggesting that increased human encroachment, human-mediated disease spread, and further reductions in gorilla genetic variation have contributed to high levels of environmental and genetic stress in *Homo sapiens*' second closest living relative.

## Data accessibility

Data and code are available from Zenodo: https://doi.org/10.5281/zenodo.5546594.
